# Corynebacterium periprosthetic joint infection: a systematic review of 52 cases at 2.5 years follow-up

**DOI:** 10.1007/s00402-023-04844-8

**Published:** 2023-03-30

**Authors:** Yannick Seutz, Henrik Bäcker, Doruk Akgün, Siegfried Adelhoefer, Philipp Kriechling, Marcos R. Gonzalez, Daniel Karczewski

**Affiliations:** 1 Department of Orthopaedic Surgery and Traumatology, Charité Berlin, University Hospital, Chariteplatz 1, 10117 Berlin, Germany; 2 Department of OrthopaedicsBalgrist University Hospital, University of Zürich, Forchstrasse 340, 8008 Zurich, Switzerland; 3 Department of Orthopaedic Surgery, Musculoskeletal Oncology Service, Massachusetts General Hospital-Harvard Medical School, 55 Fruit Street, Boston, MA 02114 USA

**Keywords:** Difficult to treat pathogens, Hip infection, Knee infection, Elbow infection, Shoulder infection, Gram-positive PJI, Atypical pathogens

## Abstract

**Introduction:**

While large progress has been achieved in identifying and treating the most common pathogens involved in periprosthetic joint infections (PJI), there remains limited knowledge on atypical pathogens such as Corynebacterium. For that reason, we analyzed infection and diagnostical characteristics, as well as treatment outcome in Corynebacterium PJI.

**Methods:**

A systematic review was performed based on a structured PubMed and Cochrane Library analysis using the PRISMA algorithm. The search was performed by 2 independent reviewers, and articles from 1960 to 2022 considered eligible for inclusion. Out of 370 search results, 12 studies were included for study synthesis.

**Results:**

In total, 52 cases of Corynebacterium PJI were identified (31 knees, 16 hips, 4 elbows, 1 shoulder). Mean age was 65 years, with 53% females, and a mean Charlson Comorbidity Index of 3.9. The most common species was Corynebacterium striatum in 37 cases (71%). Most patients were treated with two-stage exchange (40%), isolated irrigation and debridement (21%), and resection arthroplasty (19%). Mean duration of antibiotic treatment was 8.5 weeks. At a mean follow-up of 2.5 years, there were 18 reinfections (33%), and 39% were for Corynebacterium. Initial infection by Corynebacterium striatum species was predictive of reoperation (*p* = 0.035) and reinfection (*p* = 0.07).

**Conclusion:**

Corynebacterium PJI affects multimorbid and elderly patients, with one in three developing a reinfection at short term. Importantly, the relative majority of reinfections was for persistent Corynebacterium PJI.

## Introduction

Periprosthetic joint infections (PJIs) remain a devastating complication following arthroplasty [1, 2]. Although large progress was made in identifying the most common pathogens involved, such as Staphylococcus aureus in acute infections, as well as coagulase-negative Staphylococci (CNS) in chronic infections, there is limited knowledge on characteristics of atypical pathogens such as Candida or gram-negative bacteria [3, 4].

Gram-positive bacilli are an atypical cause of PJI and often considered a contaminant in microbiology findings. The gram-positive bacillus Corynebacterium spp. is a facultatively anaerobically growing, gram-positive rod, and part of the standard flora of human skin and mucosa [3, 4]. Given its primary consideration as a contaminant in the context of PJI, as well as difficulties in cultivation, and oftentimes missing standardized diagnostical tools for assessment, there are limited reports on PJI caused by Corynebacterium [3–5].

As such, this systematic review aimed to summarize all cases of Corynebacterium PJI, diagnostical and clinical characteristics, as well as possible treatment strategies, and reported outcomes.

## Material and methods

A systematic review was performed based on a structured PubMed and Cochrane Library analysis using the PRISMA (Preferred Reporting Items for Systematic Reviews and Meta-Analyses) criteria. Search terms were as followed: “Corynebacterium PJI OR Corynebacterium periprosthetic joint infection OR Corynebacterium joint infection OR Corynebacterium hip infection OR Corynebacterium knee infection OR Corynebacterium shoulder infection OR Corynebacterium elbow infection OR Corynebacterium finger infection OR Corynebacterium foot infection OR Corynebacterium septic arthritis OR Corynebacterium osteomyelitis “.

Final inclusion criteria were: (1) studies published from 1960 to September 2022, (2) PJI caused by Corynebacterium, and (3) clinical and diagnostical case description as well as an available follow-up. Exclusion criteria were: (1) non arthroplasty infections (soft tissue, osteosynthesis material, native joints, osteomyelitis), (2) animal and experimental studies without patients, (3) Corynebacterium PJI in the context of new diagnostical tests without detailed clinical follow-up and case description, and (4) Corynebacterium PJI as part of larger PJI cohorts or combined with other infection types without a detailed and separate description of the Corynebacterium PJI. Although cases of osteomyelitis and septic arthritis in native joints without a prosthesis in situ were excluded in the study, search criteria included these terms as older studies partially used a different terminology than PJI.

**Fig. 1:**
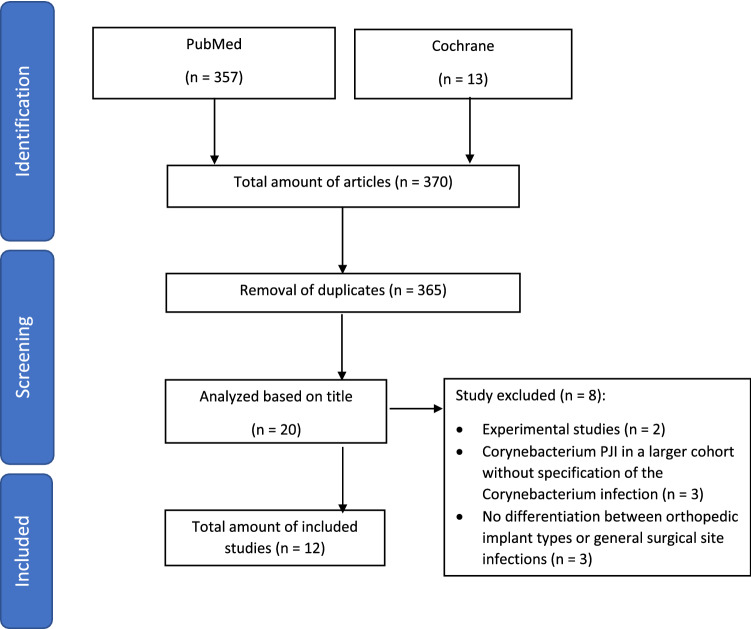
PRISMA Flowchart

Search was divided into two phases (Fig. 1). Phase one included the identification of publications using the upper search terms. In phase two, screening of abstract was performed based on the eligibility criteria of this study. The search was performed by two independent reviewers (YS, DK). Outcome parameters included infection characteristics (affected joint, corynebacterium species, coexisting microbes), patient characteristics (age, sex, Body Mass Index (BMI), Charlson Comorbidity Index (CCI) [6], secondary diseases), arthroplasty details (year and indication for primary implantation), diagnostical work-up (CRP, ESR, preoperative joint aspiration, intraoperative tissue samples, symptoms, histopathology), surgical and antibiotic treatment, as well as outcome evaluation (follow-up, reinfection rates, revision rates, perioperative complications, mortality).

Statistical analysis was performed using SPSS (SPSS Inc., Chicago, IL, USA), with T- and Mann–Whitney U tests for continuous variables, as well as Fisher exact test for categorical variables. A *p*-value < 0.05 was considered significant whereas a *p*-value < 0.1 was interpreted as a trend to significance.

## Results

In total, 370 studies were identified using the aforementioned search terms. After exclusion of duplicates, 365 titles were screened for study inclusion, and 20 articles analyzed in detail. Of these 20 studies, 2 investigations were excluded as they were experimental studies without clinical details [7, 8], 3 studies as they reported of Corynebacterium PJI as part of a larger epidemiological cohort without specification of the Corynebacterium PJI itself [9–11], and another 3 as they were not differentiating between orthopedic implant types or referred to general surgical site infections without implants [12–14].

As such, a total of 12 studies with 52 cases of Corynebacterium PJI were included. Among these, there were 31 total knee arthroplasties (TKAs), 16 total hip arthroplasties (THAs), 4 elbow prosthesis and one shoulder prosthesis (Table 1) [15–26]. Cases were reported from North America, Europe and Asia between 1994 and 2022. The pathogen spectrum was as followed: Corynebacterium striatum (37 cases), Corynebacterium jeikeium (8 cases), Corynebacterium amycolatum (2 cases), Corynebacterium bovis (1 case) and unspecified species (4 cases). Mean age at time of Corynebacterium PJI was 64.7 years (± 12.1). Two studies did not clarify the patient’s sex. Among the remaining 10 studies, 19 were females, and 17 were males. Rheumatoid arthritis (29%) and diabetes mellitus type II (25%) were the most common secondary diseases. Mean Charlson Comorbidity Index (CCI) was 3.9 (range 0–11). A total of 6 cases had a prior PJI, none for Corynebacterium.

**Table 1:** Included cases of Corynebacterium PJI

Study	Weller et al. [15], 1994 Case 1	Case 2	Yildiz S et al. [16], 1995	Tleyjeh et al. [17], 2005	Achermann et al. [18], 2009	Wee et al. [19], 2013	Streubel et al. [20], 2016 Case 1	Case 2	Case 3	Ferry et al. [21], 2017	Fernández-Esgueva et al. [22], 2019	Hernandez et al. [23], 2020 Case 1	x Case 2	x Case 3	Case 4
Country, Region	U.K., Oxford	U.K., Oxford	Turkey, Ankara	USA, Rochester	Switzerland, Zurich	Singapore, Singapore	USA, Omaha	USA, Omaha	USA, Omaha	France, Lyon	Spain, Zaragoza	USA, Durham	USA, Durham	USA, Durham	USA, Durham
PJI type	Hip	Hip	Knee	Hip	Shoulder	Elbow	Elbow	Elbow	Elbow	Knee	Knee	Hip	Hip	Hip	Knee
Age (years)	52	44	67	78	62	67	39	61	54	54	85	69	77	72	63
Sex	Female	Male	Female	Male	Female	Female	Female	Female	Male	NA	Female	Female	Female	Female	Male
BMI (kg/m2)	NA	NA	NA	NA	NA	NA	NA	NA	NA	NA	NA	30	37	36.13	40
CCI	NA	NA	NA	NA	NA	NA	1	NA	1	2	6	3	3	4	5
Secondary diseases	NA	NA	NA	Multiple	NA	NA	Rheumatoid arthritis	NA	Post-traumatic arthritis	Hemophilia, hepatitis C	Hypertension, chronic atrial fibrillation, asthma, breast cancer	Rheumatoid arthritis	Atrial fibrillation, bilateral TKA	Rheumatoid arthritis	Diabetes, stroke, tobacco use
Primary implantation (PI)	1986	1974	1988	2002	2007	NA	NA	NA	NA	NA	2000	2017	2011	2012	2015
Indication for PI	NA	Osteoarthritis	Osteoarthritis	NA	Secondary arthrosis	NA	NA	NA	NA	NA	Osteoarthritis	NA	NA	NA	Osteoarthritis
Year of initial PJI	1992	1992	1994	2002	2008	NA	NA	NA	NA	2015	2016	2017	2013	2015	2018
Microbe identified in PJI prior to Corynebacterium PJI	NA	NA	NA	NA	NA	NA	NA	NA	NA	Klebsiella pneumoniae, Staphylococcus aureus	-	NA	NA	E. coli, VRE, Proteus vulgaris, Serratia marcescens	NA
Year of initial Corynebacterium PJI	1992	1992	1994	2004	2008	NA	NA	NA	NA	2016	2016	2017	2019	2018	2018
Corynebacterium species	C. jeikeium	C. jeikeium	C. jeikeium	C. jeikeium	C. bovis	C. spp.	C. spp.	C. spp	C. spp	C. striatum	C. striatum	C. striatum	C. striatum	C. striatum	C. striatum
Coexisting microbe/Polymicrobial	None	None	None	CNS	None	None	NA	NA	MSSA, MRSA, CNS	Enterobacter asburiae	None	None	Mycobacterium avius	None	None
Onset type	Chronic onset	Chronic onset	Chronic onset	Chronic onset	Chronic onset	NA	NA	NA	NA	Chronic onset	Chronic onset	Acute onset	Chronic onset	Chronic onset	Chronic onset
Initial diagnosis	Tissue samples	Tissue samples	Joint aspiration	Joint aspiration	Joint aspiration	Tissue samples	Tissue samples	Tissue samples	Tissue samples	Tissue samples	Joint aspiration	Tissue samples	Tissue samples	Tissue samples	Tissue samples
CRP	NA	NA	NA	High CRP	0.7 mg/dl	NA	NA	NA	NA	NA	10.93 mg/dl	24 mg/dl	12.71 mg/dl	17.9 mg/dl	1.91 mg/dl
ESR	NA	NA	NA	High ESR	22 mm/hr	NA	NA	NA	NA	NA	NA	100 mm/hr	90 mm/hr	108 mm/hr	100 mm/hr
Leading symptoms	Pain, persistent discharging sinus tract	Sinus tract	Disabling pain	Local inflammation signs	Pain, stiffness	NA	NA	NA	NA	Bloody discharge	Local signs of inflammation, pain, limited flexion	Local inflammation signs	Local inflammation signs, draining sinus tract	Sinus tract	15-degree flexion contracture
Intraoperative histopathology	Acute and chronic inflammatory changes	NA	NA	Krenn and Morawitz II	Krenn and Morawitz III	No acute inflammation	NA	NA	NA	NA	NA	NA	NA	NA	NA
Initial surgical treatment for Corynebacterium PJI	Two-stage exchange	Two-stage exchange	Two-stage exchange	I&D, revision of the acetabular component	Two-stage exchange	None (identified during revision)	Two times I&D	Two times I&D	Two times I&D	None, instead antibiotic treatment (due to high risk of potential bleeding); After 3 months resection	NA	DAIR	Two-stage exchange	I&D	Two-stage exchange
Prosthesis reimplantation/ prosthesis in situ	Yes	Yes	Yes	Yes	Yes	Yes	Yes	Yes	Yes	No	Yes	No	Yes	No	Yes
Total treatment for Corynebacterium PJI	Vancomycin 42 days	Vancomycin 42 days	Vancomycin 35 days + Tetracycline 56 days	Vancomycin 42 days	Imipenem, followed by oral Amoxicillin 84 days	Cephalexin 14 days	Amoxicillin Duration NA	Vancomycin Duration NA	Vancomycin Duration NA	Vancomycin + Imipenem + Fosfomycin 84 days	Vancomycin + Ceftazidime 5 days; later 9 days i.v. Linezolid	Vancomycin 42 days	Vancomycin + Ertapenem 56 days	Tedizolid (Vancomycin resistant organism) 56 days	Vancomycin 42 days
Outcome of initial treatment	No clinical signs of infection	No clinical signs of infection	No clinical signs of infection	No clinical signs of infection	No clinical signs of infection	No clinical signs of infection	Reinfection without pathogen detection I&D	Periprosthetic fracture treated with open reduction and internal fixation	No clinical signs of infection	New infection due to E. asburiae Resection arthroplasty	No clinical signs of infection	Reinfection due to C. striatum Resection arthroplasty	Dislocation (subsequent infection; couned as aseptic revision)	Reinfection due to C. striatum and E. faecium I&D	No clinical signs of infection
Follow-up	12 months	12 months	7 months	6 months	2.5 months	67 months	72 months	204 months	24 months	9 months	NA	30 months	9 months	14 months	13 months
Perioperative complications	NA	NA	None	None	Central bone necrosis of the humerus	NA	None	None	Complex regional pain syndrome	Hematoma with bloody discharge, hemorrhagic discharge	None	None	None	None	None
Death by PJI	No	No	No	No	No	No	No	No	No	No	No	No	No	No	No

Study	Case 5	Case 6	Streifel et al. [24], 2022	Tabaja et al. [25], 2022 Case 1	Case 2	Case 3	Case 4	Case 5	Case 6	Case 7	Case 8	Case 9	Case 10	Case 11	Case 12
Country, Region	USA, Durham	USA, Durham	USA, Portland	USA, Rochester	USA, Rochester	USA, Rochester	USA, Rochester	USA, Rochester	USA, Rochester	USA, Rochester	USA, Rochester	USA, Rochester	USA, Rochester	USA, Rochester	USA, Rochester
PJI type	Knee	Knee	Knee	Knee	Knee	Knee	Knee	Hip	Knee	Knee	Knee	Knee	Knee	Knee	Hip
Age (years)	70	66	65	79	91	60	82	40	59	75	58	65	50	59	63
Sex	Female	Male	Male	Male	Male	Male	Female	Female	Male	Male	Female	Female	Male	Male	Female
BMI (kg/m2)	28.8	24.13	NA	25.8	32.2	32.3	36	34.6	62	36.4	29.8	37	21.9	42.4	20
CCI	5	4	NA	3	6	3	11	0	2	3	3	9	2	4	3
Secondary diseases	Rheumatoid arthritis, diabetes, hypoalbuminemia	Rheumatoid arthritis, olecranon septic bursitis	NA	Coronary artery disease, chronic left lower extremity ulcer	Chronic kidney disease	Diabetes	Chronic artery disease, diabetes, congestive heart failure, chronic kidney disease, peripheral artery disease, Hodgkin-lymphoma	Teratologic hip dislocation	Congestive heart failure	Chronic artery disease	Chronic kidney disease	Liver cirrhosis, diabetes, congestive heart failure, chronic right lower extremity ulcer, chronic kidney disease	Klipperl-trenaunay syndrome, asplenia	Coronary artery disease, congestive heart failure, chronic kidney disease, chronic left lower extremity ulcer	Rheumatoid arthritis, morbus Crohn, immunosuppressive medications
Primary implantation (PI)	2018	2015	1980	NA	NA	NA	NA	NA	NA	NA	NA	NA	NA	NA	NA
Indication for PI	Osteoarthritis	NA	NA	NA	NA	NA	NA	NA	NA	NA	NA	NA	NA	NA	NA
Year of initial PJI	2018	NA	2008	NA	NA	NA	NA	NA	NA	NA	NA	NA	NA	NA	NA
Microbe identified in PJI prior to Corynebacterium PJI	-	Pseudomonas aeruginosa, Staphylococcus warneri, Staphylococcus epidermidis	Stpahylococcus hominis, Dermacoccus spp.	NA	NA	NA	NA	NA	NA	NA	NA	NA	NA	MSSA	NA
Year of initial Corynebacterium PJI	2018	2018	2008	NA	NA	NA	NA	NA	NA	NA	NA	NA	NA	NA	NA
Corynebacterium species	C. striatum	C. striatum	C. striatum	C. striatum	C. striatum	C. striatum	C. striatum	C. amycolatum	C. jeikeium	C. striatum	C. striatum	C. striatum	C. jeikeium	C. striatum	C. striatum
Coexisting microbe/Polymicrobial	Peptoniphilus asaccharolyticus, Prevotella bivia	None	Staphylococcus haemolyticus, Staphylococcus epidermidis	NA	NA	NA	NA	NA	NA	NA	NA	NA	NA	NA	NA
Onset type	Acute onset	Chronic onset	Chronic onset	Chronic onset	Chronic onset	Acute onset	Chronic onset	Acute onset	Chronic onset	Chronic onset	Chronic onset	Chronic onset	Chronic onset	Chronic onset	Chronic onset
Initial diagnosis	Tissue samples	Tissue samples	Tissue samples	Tissue samples	Tissue samples	Tissue samples	Tissue samples	Tissue samples	Tissue samples	Tissue samples	Tissue samples	Tissue samples	Tissue samples	Tissue samples	Tissue samples
CRP	35 mg/dl	43.9 mg/dl	NA	NA	NA	NA	NA	NA	NA	NA	NA	NA	NA	NA	NA
ESR	116 mm/hr	65 mm/hr	NA	NA	NA	NA	NA	NA	NA	NA	NA	NA	NA	NA	NA
Leading symptoms	Wound dehiscence, purulent drainage, fever	Sinus tract, non-healing wound	Persistent drainage	NA	NA	NA	NA	NA	NA	NA	NA	NA	NA	NA	NA
Intraoperative histopathology	NA	NA	NA	NA	NA	NA	NA	NA	NA	NA	NA	NA	NA	NA	NA
Initial surgical treatment for Corynebacterium PJI	DAIR	Resection arthroplasty	I&D	DAIR	DAIR	DAIR	DAIR	DAIR	DAIR	DAIR	Two-stage exchange	Two-stage exchange	Two-stage exchange	Two-stage exchange	Two-stage exchange
Prosthesis reimplantation/ prosthesis in situ	No	No	Yes	Yes	Yes	Yes	Yes	Yes	Yes	Yes	Yes	Yes	Yes	Yes	Yes
Total treatment for Corynebacterium PJI	Vancomycin + Piperacillin-Tazobactam 14 days	Vancomycin 42 days	Daptomycin 33 days, Linezolid 2 days	Doxycycline 350 days	Amoxicillin + Minocycline 14 days	49 days Vancomycin + 91 days minocycline	Vancomycin 49 days	Daptomycin 28 days	Daptomycin + Ertapenem 42 days	Vancomycin 42 days	Vancomycin 42 days	NA	Vancomycin 42 days	Vancomycin 42 days	Vancomycin + Ertapenem 98 days
Outcome of initial treatment	Reinfection due to C. striatum Resection arthroplasty	Above knee amputation without pathogen detection	Reinfection due to C. striatum DAIR	Reinfection due to C. striatum Two-stage exchange	No clinical signs of infection	No clinical signs of infection	No clinical signs of infection	No clinical signs of infection	Chronic sinus tract with culture negative DAIR	No clinical signs of infection	No clinical signs of infection	Reinfection with culture negative DAIR	No clinical signs of infection	No clinical signs of infection	No clinical signs of infection
Follow-up in months	11 months	12 months	12 months	12 months	7 months	18 months	4 months	23 months	6 months	11 months	79 months	24 months	75 months	39 months	15 months
Perioperative complications	Increased left lower extremity pain and darkening of her toes	None	NA	NA	NA	NA	NA	NA	NA	NA	NA	NA	NA	NA	NA
Death by PJI	Yes	No	No	No	No	No	No	No	No	No	No	No	No	No	No

Study	Case 13		Case 14		Case 15		Case 16		Case 17		Case 18		Case 19		Panuu et al. [26], 2022 Case 1—3		Case 4		Case 5		Case 6—7		Case 8—10		Case 11—14		Case 15	
Country, Region	USA, Rochester		USA, Rochester		USA, Rochester		USA, Rochester		USA, Rochester		USA, Rochester		USA, Rochester		USA, Cleveland		USA, Cleveland		USA, Cleveland		USA, Cleveland		USA, Cleveland		USA, Cleveland		USA, Cleveland	
PJI type	Knee		Hip		Knee		Hip		Knee		Hip		Knee		Hip		Hip		Hip		Knee		Knee		Knee		Knee	
Age (years)	55		69		68		57		85		69		65		NA													
Sex	Female		Male		Female		Male		Male		Female		Male		NA													
BMI (kg/m2)	25.5		32		33.5		31.3		27.9		26.1		42.8		NA													
CCI	2		NA		3		5		7		7		3		NA													
Secondary diseases	Rheumatoid arthritis, marfan syndrome, immunosuppressive medications		NA		Rheumatoid arthritis, Sjogren syndrome		Diabetes, chronic kidney disease, COPD, ataxia		Coronary artery disease, diabetes, colon carcinoma		Peripheral artery disease, chronic kidney disease, localized bladder carcinoma		Diabetes, chronic inflammatory demyelinating polyneuropathy, immunosuppressive medications		NA													
Primary implantation (PI)	NA		NA		NA		NA		NA		NA		NA		NA													
Indication for PI	NA		NA		NA		NA		NA		NA		NA		NA													
Year of initial PJI	NA		NA		NA		NA		NA		NA		NA		NA													
Microbe identified in PJI prior to Corynebacterium PJI	NA		NA		NA		NA		NA		NA		NA		NA													
Year of initial Corynebacterium PJI	NA		NA		NA		NA		NA		NA		NA		NA													
Corynebacterium species	C. jeikeium		C. amycolatum		C. jeikeium		C. striatum		C. striatum		C. striatum		C. striatum		C. striatum													
Coexisting microbe/Polymicrobial	NA		NA		NA		NA		NA		NA		NA		NA													
Onset type	Chronic onset		Chronic onset		Chronic onset		Chronic onset		Chronic onset		Chronic onset		Chronic onset		NA													
Initial diagnosis	Tissue samples		Tissue samples		Tissue samples		Tissue samples		Tissue samples		Tissue samples		Tissue samples		NA													
CRP	NA		NA		NA		NA		NA		NA		NA		NA													
ESR	NA		NA		NA		NA		NA		NA		NA		NA													
Leading symptoms	NA		NA		NA		NA		NA		NA		NA		NA													
Intraoperative histopathology	NA		NA		NA		NA		NA		NA		NA		NA													
Initial surgical treatment for Corynebacterium PJI	Two-stage exchange		Two-stage exchange		Two-stage exchange		Two-stage exchange		Resection arthroplasty with spacer insertion		Resection arthroplasty with residual cerclage wires		Resection arthroplasty with spacer insertion		Two-stage exchange		Resection with spacer insertion		Girdlestone		Resection with spacer insertion		Two-stage exchange		I&D		I&D	
Prosthesis reimplantation/ prosthesis in situ	Yes		Yes		Yes		Yes		No		No		No		Yes		No		No		No		Yes		Yes		No	
Total treatment for Corynebacterium PJI	Vancomycin 42 days		Vancomycin 42 days		daptomycin + Ertapenem 42 days		Vancomycin 42 days		Vancomycin 56 days		Meropenem 56 days		Vancomycin 42 days		Combination of Vancomycin, Cephalosporines, Penicillin, Beta-lactam, Doxycycline, Carbapenems, Fluocinolones 81.4 ± 97.8 days		Combination of Vancomycin, Aminoglycosides, Carbapenems, Cephalosporine, Doxycycline 50 days		Combination of Piperacillin-Tazobactam, Vancomycin. Cephalosporines 40 days		Combination of Vancomycin, Cephalosporines, Carbapenems, Penicillin 26.5 ± 7.8 days		Combination of Vancomycin, Cephalosporines, Daptomycin, Penicillin, Beta-lactam, Fluconazole (1 case) 71.3 ± 70.3 days		Combination of Vancomycin, Cephalosporines, Penicillin + Beta-lactam, Daptomycin, Doxycycline, Fluorchinolones 27 ± 32.1 days		Combination of Daptomycin, Doxycycline, Rifampin, Ciprofloxacin, Linezolid 172 days	
Outcome of initial treatment	No clinical signs of infection		No clinical signs of infection		No clinical signs of infection		No clinical signs of infection		No clinical signs of infection		No clinical signs of infection		No clinical signs of infection		New infection due to: Case 1: VRE, E. faecium, C. albicans I&D Case 2: Proteus mirabilis DAIR Case 3: VRE Resection arthroplasty		New infection due to MRSA I&D		No clinical signs of infection		Case 6: New infection due to VRE Above knee amputation Case 7: No clinical signs of infection		Case 8: Infection with culture negative Revision arthroplasty Case 9–10: No clinical signs of infection		Case 11: Reinfection due to C. striatum Above knee amputation Case 12–14: No clinical signs of infection		Reinfection due to C. striatum and Enterobacter cloacae Resection arthroplasty	
Follow-up in months	6 months		67 months		15 months		55 months		29 months		54 months		31 months		33.7 ± 24.2 months		17.4 months		42.5 months		34.9 ± 14 months		11.7 ± 1.9 months		41.9 ± 22.8 months		7 months	
Perioperative complications	NA		NA		NA		NA		NA		NA		NA		NA													
Death by PJI	No		No		No		No		No		No		No		No		No		No		No		No		No		No	

The initial diagnosis of Corynebacterium PJI was confirmed via intraoperative tissue samples in 33, and preoperative joint aspiration in 4 cases. Panuu et al. [26] did not clarify the methods used for initial diagnosis (15 cases). A polymicrobial Corynebacterium PJI was identified in 6 cases, with co-existing pathogens including Mycobacterium avius, Peptoniphilus asaccharolyticus, Prevotella bivia, Enterobacter asburiae, Staphylococcus aureus as well as multiresistant Staphylococcus aureus (MRSA) and CNS. Among 33 cases with a description of symptom onset, 29 were of chronic entity (symptoms longer than 4 weeks), whereas 4 were acute infections (shorter than 4 weeks). Mean preoperative CRP was 18.4 mg/dl (± 15.3), mean ESR was 85.88 mm/hr (± 32.5).

Surgical treatment included debridement, antibiotics and implant retention with exchange of mobile components (DAIR) in 9 joints, permanent resection arthroplasty in 10 cases, isolated irrigation and debridement (I&D) in 11 cases, and two-stage exchange in 21 patients. One Corynebacterium PJI was identified incidentally in the course of an elbow revision due to loosening, but considered PJI by the authors, as two positive intraoperative samples of Corynebacterium spp. were found.

The mean duration of antibiotic treatment was 8.5 weeks (± 8.1 weeks). The most common antibiotic groups used were Vancomycin (75%), Cephalosporines (31%), Penicillin with beta-lactam antibiotics (31%), Carbapenems (27%), and Tetracyclines (25%).

Mean follow-up was 30.1 months (± 35 months). There were 20 reoperations, including one for periprosthetic fracture requiring open reduction and internal fixation, one for dislocation treated with open reduction and head exchange, and 18 for reinfections. Mortality was low, with one patient dying from sepsis at 11 months. A total of 4 nonoperative complications occurred, including knee hematoma, chronic pain syndrome, and darkening of the toes in 3 TKAs, as well as one central humerus necrosis in case of a shoulder PJI.

Treatment for the 18 reinfections included DAIR in 4 cases, resection arthroplasty with spacer insertion in 4 cases, irrigation and debridement in 4 cases, above knee amputation in 3 cases, spacer exchange in one case, unspecified revision arthroplasty with extensor mechanism reconstruction in one case, and two-stage exchange in another case. In total, there were 7 reinfections by Corynebacterium striatum, including 2 mixed infections with Enterococcus faecium and Enterobacter cloacae, 6 reinfections by a different pathogen (Enterobacter asburiae; Enterococcus faecium and Candida albicans; Proteus mirabilis; Vancomycin Resistant Enterococcus (VRE); VRE; MRSA), and 5 culture negative cases.

The most common Corynebacterium species, Corynebacterium striatum, demonstrated a significantly higher rate of reoperations when compared to other Corynebacterium species (*p* = 0.035), and trended towards increased rates of reinfection (*p* = 0.07). The remaining baseline and surgical characteristics including age, CRP, ESR, presence of coexisting pathogens, duration of antibiotic treatment, and follow-up did not show a statistically significant difference among species (Table 2).

**Table 2:** Subanalysis depending on Corynebacterium species

Parameters	C. striatum	Other C. spp.	P-value
Joints (n)	37	11	-
Age in years (mean)	70	59	0.588
CRP in mg/dl (mean)	21	1	0.127
ESR in mm/hr (mean)	97	22	0.13
Coexisting pathogen (n)	4	1	0.99
Duration of total antibiotic treatment in days (mean)	65	49	0.662
Perioperative complications (n)	2	1	0.551
Follow-up in months (mean)	24	21	0.146
Reoperation (n)	17	1	0.035
Reinfection (n)	16	1	0.070

Cases without specification of C. spp. were excluded for subanalysis

## Discussion

Nondiphtheria Corynebacteria are widely considered an opportunistic commensal of the human skin and mucosa with little to unknown potential to cause infections [3]. As such, little attention has been paid to this bacterium in the context of PJI with a limited number of reports to date. This systematic review analyzed all existing Corynebacterium PJIs to date while including a total of 52 infections at a mean follow-up of 2.5 years. Our results demonstrated Corynebacterium PJIs to primarily affect total knee arthroplasties (60%) in old and multimorbid patients, with one in three joints developing recurrent infection at short term.

Knowledge on epidemiological characteristics is essential, as certain pathogens are known to be attributable to certain risk groups [27, 28]. Our cohort demonstrated no tendency to a certain sex, although patients were multimorbid. In fact, more than 50% of patients showed secondary diseases, with rheumatoid arthritis and diabetes mellitus being most prevalent. Moreover, the mean age of our patients was high, falling in line with previous reports on gram-positive PJIs, and representing another risk factor for PJI [29, 30]. Importantly, the knee was the most common joint affected. We believe this to be attributable to gram-positive Corynebacterium being a part of the normal skin flora, whereas gram-negative pathogens were described to more frequently affect the hip, possibly due to its proximity to the gastrointestinal tract [31].

Diagnostical work-up of Corynebacterium PJI is challenging. This is due to the fact that Corynebacterium may not be part of the standard work-up, is universal part of the human microbiome, and the identification process itself is expensive [3, 4, 32]. Our results showed most cases to be identified out of intraoperative tissue samples. As previously acknowledged, the identification of Corynebacterium spp. as commensals of the human skin and mucosa must always be considered in the context of a possible contamination before drawing a final conclusion. Importantly, only six cases were of polymicrobial nature, and CRP and ESR were significantly increased, reducing the likelihood of possible contamination.

All but 4 infections showed a chronic symptom onset. Accordingly, the majority of cases were treated without implant retentions attempts, given a high likelihood of completed biofilm formation [33]. However, not all patients were treated according to current guidelines with a substantial number of patients undergoing DAIR and/or isolated irrigation in case of chronic infections. We believe this to be associated with a multimorbid and old patient cohort being at high risk of perioperative complications in case of complete prosthesis removal and later reimplantation. In fact, Ferry et al. attempted a pure antibiotic treatment attempt given a high risk of bleeding in their patient, before deciding upon resection arthroplasty 3 months later [21].

In addition to an adequate surgical strategy, the selection of a correct antimicrobial therapy plays an essential role [34]. In our cohort, the majority of cases were treated with Vancomycin and Cephalosporines. This is important, as Corynebacterium spp., especially the jeikeium species, has shown a resistance rate of up to 60% against various groups of antimicrobials, including Aminoglycosides, Penicillin, and Cephalosporines [12]. Although no detailed resistance pattern was reported in included studies, the use of the aferomentioned antibiotics might indicate a low rate of resistance against standard antibiotics in cases of Corynebacterium PJI.

With respect to the outcome, one in three joints developed reinfection at short term. We believe this devastating outcome to be caused by a number of factors, including a substantial number of secondary diseases, an old age, as well as a number of implant retention attempts in chronic infections [35, 36]. Importantly, Corynebacterium striatum had a significantly higher rate of reinfections as opposed to other species, resulting in a failure rate of nearly 50%. While this might lead to the assumption that striatum species are a risk factor for failure in Corynebacterium PJIs, we acknowledge mean age, CRP, and ESR to be higher in this group of patients, although the effect was statistically not significant.

This systematic review had several limitations that were primarily attributable to its included studies. Foremost, we included a small number of cases with inconsistent information, resulting in a highly heterogenous group of patients. Moreover, PJI among 4 different joint types was inconsistently defined between studies, and treatment occurred over nearly 3 decades with substantially different strategies used. Finally, our results represent short-term outcomes only.

In conclusion, Corynebacterium PJI is a rare, yet severe complication occurring in the elderly and multimorbid, while resulting in significant treatment failures. One in five patients will experience Corynebacterium persistence at short term. Further studies will be necessary to draw additional conclusions on the midterm outcomes, as well as the role of the different species involved.

## Data Availability

Does not apply.
